# The Vibrating Helmet

**Published:** 1892-11-05

**Authors:** 


### CONTEMPORARY THEORIES AND RESEARCH.

### THE VIBRATING HELMET

In *La Nouvelle Revue* M. Meunier gives an interesting account of the treatment of paralysis and nervous disorders by a system of electric vibration. Struck by the benefit derived by paralytic patients from long journeys by rail or carriage, M. Charcot invented an easy chair to which a constant movement was communicated by an electric current. He found that the more vigourously the patient was shaken, the better he felt, and that after a quarter of an hour's sitting he was a different creature. The perfecting of the method however has been left to Dr. Gilles de la Tourette, a pupil of M. Charcot. He has succeeded in constructing a kind of vibrating helmet, said to be infallible for "migraine," sleeplessness, and other nervous disorders. It consists of a head piece framed of light steel bars, into which the head fits closely, and it is supplied with an electric motor revolving 600 times a minute. At each revolution a uniform vibration is conveyed to the metal bands and thence to the head which they enclose. It seems the sensation is not disagreeable; it induces a kind of stupor to which the whirring of the machine contributes. At the end of a few minutes the patient experiences a general lassitude and inclination to sleep productive of the most salutary results.

